# Diagnostic precision and identification of rare diseases is dependent on distance of residence relative to tertiary medical facilities

**DOI:** 10.1186/s13023-021-01769-6

**Published:** 2021-03-22

**Authors:** Anna-Lena Walter, Florent Baty, Frank Rassouli, Stefan Bilz, Martin Hugo Brutsche

**Affiliations:** 1 Lung Center, Cantonal Hospital St. Gallen, Rorschacher Strasse 95, 9007 St. Gallen, Switzerland; 2 Center for Rare Diseases of Eastern Switzerland (ZSK-O), Cantonal Hospital St. Gallen, Rorschacher Strasse 95, 9007 St. Gallen, Switzerland

**Keywords:** Rare diseases, Diagnostic diversity, Distance to center

## Abstract

**Background:**

Diagnostic precision and the identification of rare diseases is a daily challenge, which needs specialized expertise. We hypothesized, that there is a correlation between the distance of residence to the next tertiary medical facility with highly specialized care and the diagnostic precision, especially for rare diseases.

**Results:**

Using a nation-wide hospitalization database, we found a negative association between diagnostic diversity and travel time to the next tertiary referral hospital when including all cases throughout the overall International Classification of Diseases version 10 German Modification (ICD-10-GM) diagnosis codes. This was paralleled with a negative association of standardized incidence rates in all groups of rare diseases defined by the Orphanet rare disease nomenclature, except for rare teratologic and rare allergic diseases.

**Conclusion:**

Our findings indicate a higher risk of being mis-, under- or late diagnosed especially in rare diseases when living more distant to a tertiary medical facility. Greater distance to the next tertiary medical facility basically increases the chance for hospitalization in a non-comprehensive regional hospital with less diagnostic capacity, and, thus, impacts on adapted health care access. Therefore, solutions for overcoming the distance to specialized care as an indicator of health care access are a major goal in the future.

## Background

More than 20 years ago the European Union (EU) identified rare diseases, defined as life-threatening or chronically debilitating conditions affecting not more than 5/10′000 persons in the community [1], as a significant problem regarding expertise, research and development of medications [2] as they were often diagnosed late [3–5], misdiagnosed or not diagnosed at all [6]. Although each single rare disease only affects a relatively small number of patients and families, due to the large number of rare diseases all together they must be considered as a rather frequent problem, thus, representing a relevant burden for health care systems worldwide. The need to bring together expertise and make efficient use of the limited available resources means that rare diseases are an area where cooperation between health care regions can add particular value [6]. Specialized centers are therefore needed as a critical factor for assuring the quality of medical care in this group of patients. The accessibility of those tertiary referral centers is crucial, as shown for different rare conditions [7, 8], but also for diseases such as cancer [9], common respiratory diseases like asthma or chronic obstructive pulmonary disease (COPD) [10] as well as cardiovascular [11] and neurological diseases [12]. This accessibility of medical care depends on different determinants, which include geographic, regulatory and socio-economic factors [13, 14] and vary depending on the health care set-up. Geographic dispersion also impacts on the management of patients. It is well known that the distance to the next hospital is arbitrative in emergencies, especially in respiratory emergencies [15] and acute myocardial infarction [11]. Krohn et al. showed that geographic dispersion leads to decreased rates of hospital discharge and a higher rate of discharge to skilled nursing facilities [16]. We hypothesized that diagnostic precision, measured by the diagnostic diversity index (DDI) as a reproducible quality indicator of the diagnostic precision of inpatient care, depending on the caseload of the hospital [17], is affected by spatial disparities along the geographical dispersion of specialized medical centers. The aim of the current project was therefore, to examine whether there is a correlation between the mean diagnostic diversity and standardized incidence rates for rare diseases from inpatient cases of residents from a particular area and their travel time to the next tertiary inpatient facility.

## Results

**Fig. 1:**
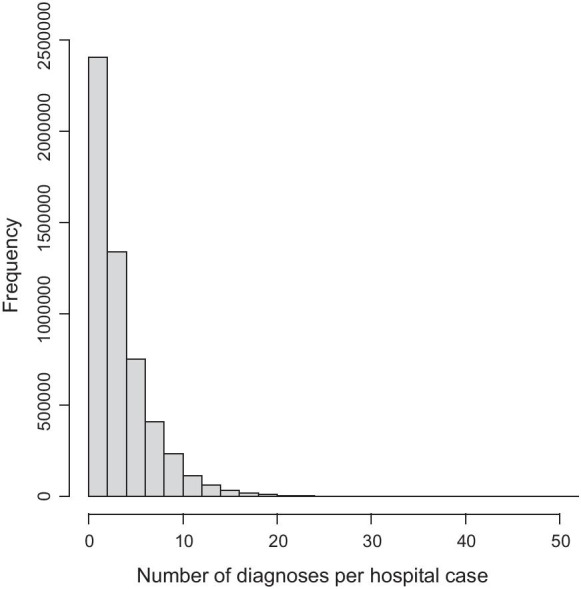
Distribution of the number of diagnoses per hospitalization case

A total of 5,390,591 inpatient cases from 2009 to 2012 were included. In these cases 17,751 different ICD-10 codes were used. Per case a median of 3 coded diagnoses (interquartile range 1–5) were observed. Figure 1 shows the distribution of the number of diagnoses. Rare disease associated ICD-10 codes were coded in 1,464,753/5,390,591 (27%) of inpatient cases.

### Association between travel time and diagnostic diversity

**Fig. 2:**
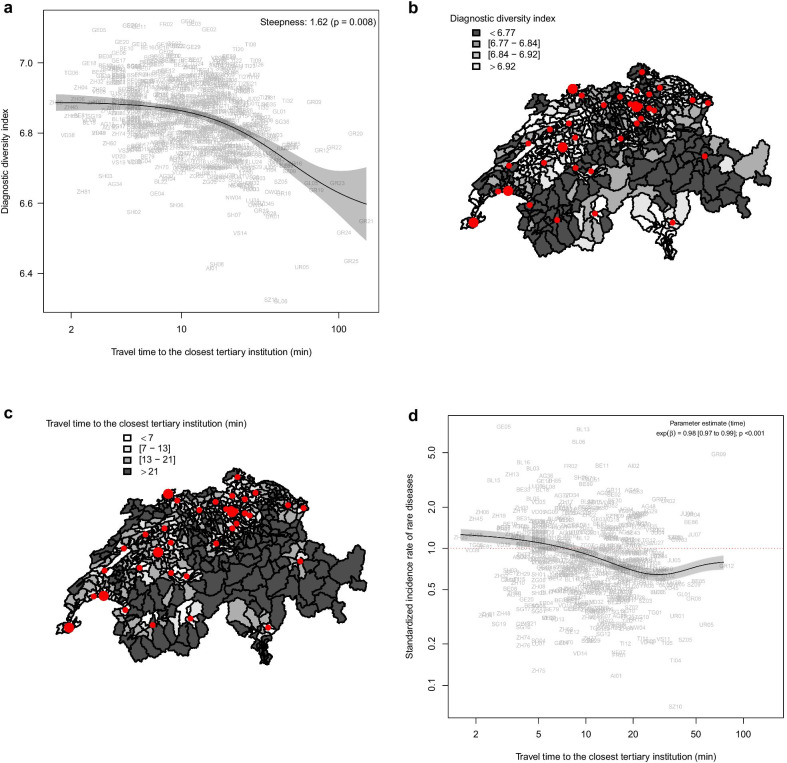
Correlation between Diagnostic Diversity Index, residence and travel time to the closest tertiary institution. **a** Diagnostic diversity of inpatient cases living in a particular residential area shows a significant inverse association with the travel time to the next tertiary hospital based on all inpatient cases overall ICD-10 codes. Inpatient cases with travel times over 20 min from their homes have a relevant proportional reduction of diagnostic diversity, typically below the mean baseline of 6.9. **b** Chloropleth of the Swiss national geographical distribution of diagnostic diversity of inpatient cases according to their Medstat-residential area and the location relative to the closest tertiary inpatient facility (Smaller red dots depict the location of the cantonal/regional tertiary institutions, whereas larger dots depict the location of university hospitals). A grey scale was chosen to depict the DDI level according to the overall quartiles of DDI. Regions with DDI higher than the third DDI quartile are depicted in white. White areas tend to co-localize with tertiary inpatient facilities. **c** Chloropleth of travel times from the individual Medstat-residential area to the next tertiary inpatient facility. A grey-scale color code based on the quartiles of the travel time to the closest tertiary inpatient facility was used. Regions located over 21 min to the closest tertiary institution are represented in dark grey. **d** The standardized incidence rate of inpatient cases living in a particular residential area diagnosed with orphan diseases in relation to travel time to the next tertiary hospital shows a significant association with a lower incidence rate for orphan diseases in more remote residential areas

Overall, based on all inpatient cases and using all ICD-10 codes, there was a significant inverse association between the diagnostic diversity and the travel time between the area of residence and the closest tertiary inpatient facility (Fig. 2a–c). The effects substantiate for travel times over 21 min, which was identified as the time where the tangent passing through the inflection point is crossing the upper asymptote.

### Association between travel time and incidence rate of rare diseases

**Fig. 3:**
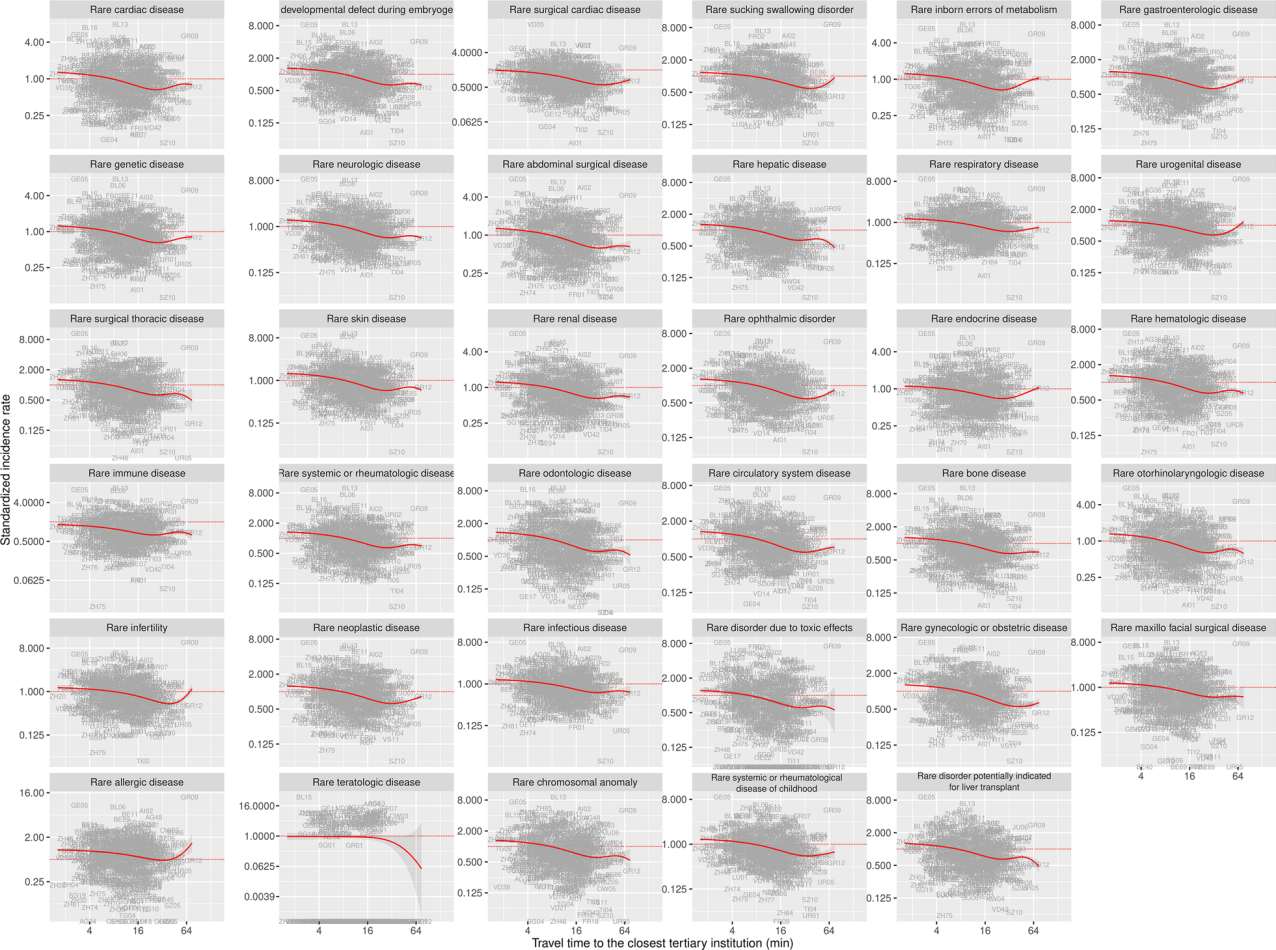
Standardized incidence of rare diseases related to travel time from residence to the next center. Overall significant associations between standardized incidence rates and travel times were observed for 33 of 35 chapters. The two exceptions were the rare disease groups “rare teratologic disease” and “rare allergic disease”. Due to data scarcity the results should be interpreted with caution particularly in these two disease groups

In order to investigate whether the above-mentioned observation of a negative association between diagnostic diversity and travel time was on the expenses of rare diseases, we restricted our analysis on rare diseases as defined by Orphanet., We found a significant inverse association between standardized incidence rates and travel time from residence to the closest tertiary inpatient institution (Fig. 2d). When analyzing rare diseases group by group a significant effect was found for all groups of rare diseases except for rare teratologic and rare allergic diseases (Fig. 3).

## Discussion

According to our study a longer travel time between residence and the next tertiary medical center is associated with less diagnostic diversity, i.e. precision, and a lower chance of being diagnosed with a rare disease, most likely due to varying accessibility to suitable health care resources even in a well-economized country like Switzerland. The effect is particularly pronounced in patients with rare diseases, where standardized incidence rates for several orphan disease groups fell below 50% at travel times of > 60 min to the next tertiary health center. Although 20–60 min travel time seems to be irrelevant in comparison to other countries. But the 39 centers are accompanied by 242 other inpatient facilities [18].

Standardized incidence rates of all different groups of rare diseases were affected except for teratologic diseases and rare allergic diseases. Teratologic diseases include embryofetopathies, especially malformation syndromes, which are rather obvious to detect. Rare allergic diseases primarily refer to many different reasons of angioedema and urticaria, which as well are visual, and, thus, less missed diagnoses regardless of the cause [19]. The term “rare diseases” is misleading as rare diseases are listed as main or co-diagnoses in more than 1 in 4 hospitalizations in our study. Of the 6172 rare diseases listed by Orphanet, 84.5% have a prevalence of < 1/1,000,000. Nguengang et al. assume that the prevalence of rare diseases is at least 3.5–5.9%, meaning 18–30 million persons in the EU, 263–446 million persons worldwide [20]. Thus, our findings point to a relevant as well as prevalent structural issue of health care accessibility in relation to geographical dispersion and might indicate a higher risk of being mis-, under- or late diagnosed in more remote residential areas. This is especially important for those rare diseases which appear accentuated in rural areas of residence as sarcoidosis [21]. Underdiagnoses or diagnostic delay has been shown to have a negative impact on the course of the disease in many rare diseases [6]. In idiopathic pulmonary fibrosis early treatment can slow down disease progression and therefore the decline in lung function [22]. Those low prevalent diseases with nonspecific symptoms as interstitial lung diseases (cough, dyspnea) are explicitly in danger to have a diagnostic delay [3, 23]. In Glucose transporter-1 deficiency syndrome ketogenic diet reduces the frequency of seizures and severity of motoric impairment [24]. Early treatment in patients with Hurler Syndrome (mucopolysaccharidosis type 1) prevents cognitive and physical disability [25]. So an early and precise diagnosis is crucial. Of course this fact is also well known in acute cardiovascular and neurological situations, but also in oncological diseases with negative impact due to therapeutic delay [26–28].

There are different possible strategies to overcome the “distance-to-center” challenge. The obvious option is to optimize health care location planning using geographic information systems, average travel distance to the next clinic site and electronic health records, as Soares et al. investigated in the United States of America (USA) [29]. Such an approach allows for a proactive planning of health care infrastructure optimizing for accessibility as well as sufficient caseload. The importance of the latter is well known for complex surgical interventions, as well as for some emergency conditions and low-risk procedures in Germany [30]. But also diagnostic performance is strongly associated with caseload [17]. In contrast to a high number of smaller hospitals with limited diagnostic capabilities, a smaller number of high-volume inpatient facilities can offer a comprehensive access to subspecialties. Tele-health care will also probably gain even more importance in the future. Tele-health care is defined as a complex intervention, with information from patients being electronically transferred over a distance to health care professionals, who analyze this information and give immediate and personalized feedback and advice to the patient [31] via telephone or internet. Electronic consultations (e-consults) “offer a rapid, direct, and documented communication pathway for consultation between primary care and specialist” [32]. Muse et al. provided an interactive platform for physicians to discuss complex cases on an international base, which found favor especially with younger medical practitioners. 37,706 physicians from 171 countries on every continent used the platform during the 2 years duration of this study [33]. As tele-health care promises to be available anywhere and anytime, it could optimize health care access independent of the geographic and socio-cultural dispersion. Furthermore there are diagnostic decision support systems (DDSSs) available to assess “case data based on incorporated medical knowledge, compiling lists of differential diagnoses appropriate for a given sample of evidence” [34]. McGowan et al. used a just-in-time librarian consultation system with a highly positive impact in decision making in primary care [35]. Electronic online-services developed by Orphanet and by other EU-funded projects are claimed to contribute to put patients in contact with other patients and develop patient communities, to share databases between research groups, to collect data for clinical research, to register patients willing to participate in clinical research, and to submit cases to experts which improves the quality of diagnoses and treatment [6]. European registries are thought to be an important column of quality of care in rare diseases. Collaborative networks between centers and smaller institutions could be another option to handle the distance-to-center problem.

## Limitations

The quality of medical coding—although professionalized in all institutions—might be limited and varying between institutions. In the current analysis, we used a mapping table provided by Orphanet, matching ORPHAcodes with ICD-10 codes. The link between ORPHAcodes and ICD-10 codes is not 1:1, and some ICD-10 codes may code for several RDs. ICD-10 codes may not adequately represent the majority of rare diseases. Nonetheless, the Orphanet mapping table was the best available approximation for the identification of RD-associated hospitalizations, and, since it was used in a consistent and systematic manner, it did not impact on the conclusions of the current work. There will be an expansion of the number of RD-specific codes in the ICD-11 nomenclature [36] which is going to come into force on January 1st, 2022 [37]. The hospitalization database does not allow accounting for differences in socio-economic status between cases or regions. Another limitation is that only inpatient cases are documented in the available data. Therefore, we concentrated on individual motorized transport times and did not consider transfer times by public transportation. Ambulances might transport patients more likely within cantonal borders than to the actual nearest hospital. Since Switzerland is a rather small country with a total area of 41,285 km^2^ [38], the longest distance to cover in our cohort was approximately 250 km. The regional differences in health care quality in larger countries are probably even more relevant depending on the distances to the next center. Besides geographic disparities there are other factors influencing access to healthcare, including e.g. the socio-economic status. Finally, pediatric hospitals were not included in the list of tertiary centers as they were not considered as general care institutions and represented an own group of 3 hospitals in Switzerland [18].

## Conclusion

The diagnostic diversity as a marker of diagnostic precision of inpatient cases was inversely related to travel time from home to the next tertiary inpatient facility—a marker for health care accessibility. The effect was particularly pronounced at travel times over 20 min, where the diagnostic diversity started to drop significantly. The effect was particularly true for rare diseases, where standardized incidence rates dropped over 50% at travel times over 60 min to the next tertiary inpatient facility. Rare diseases seem to be more likely under-, mis- and late diagnosed in individuals living more distant to a specialized referral center. A greater distance to the next tertiary medical facility basically increases the chance for a hospitalization in a non-comprehensive regional hospital with less diagnostic capacity, and, thus, impacts on adapted health care access. Thus we provide new evidence-based arguments for a proactive planning of health care infrastructure. Remote residential areas should receive more specialized medical support, e.g. through collaborative networks and/or tele-health strategies.

## Methods

### Swiss hospitalization database

We used a database provided by the Swiss Federal Office for Statistics offering a nation-wide coverage of all hospitalization cases between 2009 and 2015 (9,325,326 hospitalization cases). For each hospitalization case the data set included one main diagnosis as well as up to 50 additional diagnoses coded using the International Classification of Diseases version 10 German Modification (ICD-10-GM) codes. The data set also included the patients' area of residence, separated into 705 “Medstat” regions defined by the postal codes by MicroGIS. The distance and travel time between each region and the closest center hospital were obtained using the finaroute webtool [39]. Travel time was calculated for individual motorized transportation. The Swiss Federal Office of Health (BAG) defined 44 centers in Switzerland, including 5 university hospitals and 39 large hospitals, which are mostly cantonal hospitals [18]. The definition of a center is based on the caseload of inpatient care and advanced training categories [40], i.e. highest standard of specialized care.

### Orphanet rare disease nomenclature

Rare diseases were defined using the Orphanet rare disease nomenclature. Orphanet is a 37-country network, co-funded by the European Commission that aims to increase knowledge on RDs and improve the diagnosis, care, and treatment of people with RDs. In total, 6172 diseases [41], malformation syndromes, morphological, and biological anomalies, as well as particular clinical situations are considered as ‘rare in Europe’ [20], meaning a prevalence of 5/10,000 or less. They are divided into 35 groups [19]. In order to link the rare diseases listed by Orphanet and the cases recorded in the hospitalization database, a mapping between rare disease codes (ORPHAcodes) and ICD-10 codes provided by Orphanet was used. The cross-referencing table provided a link between 6172 ORPHAcodes and 1546 ICD-10 codes [42]. The mapping between ORPHAcodes and ICD-10 codes is not 1:1, and, in some cases, the same ICD10-code maps several ORPHAcodes. Since our hospitalization database did not include any direct coding for rare diseases, RDs were indirectly identified using ICD-10 codes. We could nevertheless assume that RD-associated ICD-10 codes provide a fair and consistent proxy for RDs.

### Statistical considerations

The diversity of diagnoses was calculated using the Shannon diversity index defined as follows:

$$H=-\sum\limits_{i=1}^{D}{p}_{i}ln\left({p}_{i}\right)$$

with D the number of diagnoses (ICD-10 codes) and *p*_*i*_ the proportional abundance of the *i*th diagnosis. Further details can be found in our previous publication [17].

The relationship between the diagnostic diversity and the travel time to the closest tertiary institution was investigated using nonlinear regression. The following 4-parameter log-logistic regression model was fitted:

$$DDI\left(t\right)={DDI}_{low}+\frac{{DDI}_{up}-{DDI}_{low}}{1+exp\left(\tau \left(t-{T}_{1/2}DDI\right)\right)}$$

with DDI(*t*) the diagnostic diversity index corresponding to the given time (*t*) to travel to the next tertiary institution; DDI_low_ and DDI_up_ the DDI lower and upper asymptotes, respectively; τthe steepness of the exponential decay as travel time increases; T_1/2_DDI the time for half decrease of DDI. The time where the tangent passing through the inflection point is crossing the upper asymptote was further derived.

Standardized incidence rates were used as a measure of relative risk, as it is commonly done in disease mapping, a sub-type of spatial analysis. For the sake of the current analysis, no spatially structured statistical models were considered. This was done in order to avoid unnecessary complexity, considering the extensive size of the current data set. The standardized incidence rate of the rare diseases in the different Swiss regions was estimated. The relationship between the standardized incidence rate and the travel time to closest tertiary institution was modeled using Poisson regression with an offset defined as the logarithm of the size of the regional population.

All analyses were done using the R statistical software [43] including the extension package “drc” for nonlinear regression [44].

## Data Availability

The Swiss Federal Office for Statistics only provides regulated access to the data for research purpose.
